# Distinct metabolomic and lipidomic profiles in serum samples of patients with primary sclerosing cholangitis

**DOI:** 10.3389/fmed.2024.1334865

**Published:** 2024-06-04

**Authors:** Tanja Fererberger, Christa Buechler, Arne Kandulski, Tanja Elger, Johanna Loibl, Stephan Schmid, Stefanie Sommersberger, Stefan Gunawan, Sebastian Zundler, Muriel Huss, Dominik Bettenworth, Sally Kempa, Simon Weidlich, Bandik Föh, Xinyu Huang, Marcin Grzegorzek, Stefanie Derer-Petersen, Ulrich L. Günther, Jens U. Marquardt, Claudia Kunst, Karsten Gülow, Martina Müller, Christian Sina, Franziska Schmelter, Hauke C. Tews

**Affiliations:** ^1^Department of Internal Medicine I, Gastroenterology, Hepatology, Endocrinology, Rheumatology, and Infectious diseases, University Hospital Regensburg, Regensburg, Germany; ^2^Department of Medicine 1, University Hospital Erlangen, Friedrich-Alexander-Universität Erlangen-Nürnberg, Erlangen-Nürnberg, Germany; ^3^Deutsches Zentrum Immuntherapie, University Hospital Erlangen, Erlangen, Germany; ^4^Department of Medicine B - Gastroenterology and Hepatology, University Hospital Münster, Münster, Germany; ^5^Practice for Internal Medicine, Münster, Germany; ^6^Department for Plastic, Hand, and Reconstructive Surgery, University Hospital Regensburg, Regensburg, Germany; ^7^Department of Internal Medicine II, School of Medicine and Health, University Hospital Rechts der Isar, Technical University of Munich, Munich, Germany; ^8^Institute of Nutritional Medicine, University Medical Center Schleswig-Holstein, Lübeck, Germany; ^9^Department of Medicine I, University Medical Center Schleswig-Holstein, Lübeck, Germany; ^10^Institute of Medical Informatics, University of Lübeck, Lübeck, Germany; ^11^Institute of Chemistry and Metabolomics, University of Lübeck, Lübeck, Germany; ^12^Fraunhofer Research Institution for Individualized and Cell-Based Medical Engineering (IMTE), Lübeck, Germany

**Keywords:** PSC, IBD, NMR, metabolome, lipidome

## Abstract

**Intoduction:**

Identification of specific metabolome and lipidome profile of patients with primary sclerosing cholangitis (PSC) is crucial for diagnosis, targeted personalized therapy, and more accurate risk stratification.

**Methods:**

Nuclear magnetic resonance (NMR) spectroscopy revealed an altered metabolome and lipidome of 33 patients with PSC [24 patients with inflammatory bowel disease (IBD) and 9 patients without IBD] compared with 40 age-, sex-, and body mass index (BMI)-matched healthy controls (HC) as well as 64 patients with IBD and other extraintestinal manifestations (EIM) but without PSC.

**Results:**

In particular, higher concentrations of pyruvic acid and several lipoprotein subfractions were measured in PSC in comparison to HC. Of clinical relevance, a specific amino acid and lipid profile was determined in PSC compared with IBD and other EIM.

**Discussion:**

These results have the potential to improve diagnosis by differentiating PSC patients from HC and those with IBD and EIM.

## Introduction

Primary sclerosing cholangitis (PSC) is a disease that typically affects intra- and/or extrahepatic bile ducts (1, 2), and can progress to end-stage liver disease with hepatic dysfunction (3). Moreover, PSC is often associated with inflammatory bowel disease (IBD) (4). An important consequence of PSC is the increased risk of malignant progression to cholangiocarcinoma (CCA), but it is also associated with higher risk for gallbladder cancer, and colorectal carcinoma (CRC) in patients with concomitant ulcerative colitis (UC) (5–7). Of note, coincidence of PSC in a patient with UC is a marked risk factor for developing colitis-associated cancer (8).

The prevalence of PSC determined by studies in Europe, North America, and Asia varies due to regional and temporal differences up to 31.7 cases per 100,000 people (Finland, 1990−2015). The latest study conducted on the prevalence detected a point prevalence of 11.0 per 1,00,000 people in December 2021 in the Faroe Islands (9).

PSC diagnosis relies on clinical symptoms, laboratory tests, imaging, and histology (10). Magnetic resonance cholangiopancreatography (MRCP) or endoscopic retrograde cholangiopancreatography (ERCP) widely confirm the diagnosis of PSC (11). MRCP is the favored procedure because it is non-invasive and can show bile ducts with high resolution (12). However, the identification of early disease stages by MRCP may exhibit lower sensitivity than performing ERCP (13). In addition to its high sensitivity, ERCP offers the advantage of therapeutic intervention, but it is more invasive than MRCP (12). Liver biopsy should only be performed in cases of unclear diagnosis, suspected small duct PSC or overlapping syndromes (12). PSC prognosis indicators encompass alkaline phosphatase (AP), perinuclear antineutrophil cytoplasmic antibodies (p-ANCA), immunoglobulin G4 (IgG4), and antiglycoprotein 2 (GP2) immunoglobulin A (IgA) (14, 15).

Environmental and genetic factors are involved in the pathogenesis of PSC (16). A marked association between PSC and the human leukocyte antigen (HLA) system as well various disease risk loci are described (17–20). Studies by Zecher et al. (21) recently discovered the new risk haplotype HLA-DP A1*02:01∼DPB1*01:01. Takeda G protein-coupled receptor (TGR)-5, which has a protective function against the toxicity of bile acid, is reduced in biliary epithelial cells in patients with PSC. Thus, the downregulation of TGR-5 may play a role in PSC pathogenesis (22).

PSC is associated with immunological alterations in concentrations of serum cytokines. High levels of interferon (IFN) gamma, interleukin (IL)-6, IL-8, IL-10 and IL-17A are described (23, 24). In addition, induction of proinflammatory T helper (TH) 17 cells toward pathogen stimulation was determined in PSC (25). Furthermore, released transforming growth factor (TGF) beta from perisinusoidal macrophages may contribute to inflammation and fibrotic process in patients with PSC (26, 27).

In summary, some data on prognosis, genetic alterations, and pathogenesis of PSC patients have been obtained in recent years (14–27). However, studies on the metabolome and lipidome of PSC patients are still a rarity (11, 28–30). We hope that our work will enable us to develop metabolomic profiles for PSC, which will increase the diagnostic accuracy of the diagnosis.

In addition, NMR spectroscopy should be used in the future to evaluate whether there are indications of which subgroups or PSC phenotypes benefit from certain therapeutic treatments. These would be steps toward a personalized treatment of PSC patients.

Metabolomics is defined as the comprehensive measurement of metabolites within a biological sample such as blood, urine, cells, or tissues. It is a common method to identify diagnostic and prognostic disease-specific biomarkers (31). In recent years, the techniques of “omics” in combination with bioinformatic methods to identify these biomarkers have been developed rapidly.

Identifying specific biomarkers can enhance the understanding of the pathogenesis and progression of the disease, guide personalized therapy, and aid in precise risk stratification (14, 32). Despite recent progress (32), the identification of PSC-specific biomarkers for diagnosis remains challenging (14, 33).

Our study aimed to analyze the metabolomic and lipidomic serum profiles of patients with PSC using nuclear magnetic resonance (NMR) spectroscopy, to identify variations in metabolite profiles. Establishing NMR spectroscopy alongside image morphology techniques in the diagnosis of PSC may sharpen the reliability of the diagnosis or even identify specific subgroups with defined risk for neoplastic progression.

## Materials and methods

### Ethical consideration

The Ethics Committee of the University of Regensburg and the University of Lübeck approved the study on 19.05.2021 (No. 21-2390-101 and No. 22-104). All study participants were informed orally as well as in writing and gave their written consent to the study participation.

### Study participants

Patients with confirmed PSC, PSC and IBD (PSC-IBD), and IBD with other extraintestinal manifestations (EIM) seen between January 2021 and December 2022 for outpatient or inpatient consultations at the Department of Internal Medicine I, University Hospital of Regensburg, were included. Controls included 40 healthy individuals recruited by the University of Lübeck, matched to the patients with PSC in terms of age, sex, and BMI. People under the age of 18, pregnant women, and people unable to give their consent were excluded.

### Sample preparation and analysis

After the collection of serum samples from 33 patients with PSC, 64 patients with EIM, and 40 healthy controls (HC), the samples were left to clot for 30 min at room temperature. Subsequently, centrifugation followed by aliquoting into samples and backup samples was performed. The samples were stored at −80°C until measurement.

Measurements were performed using proton nuclear magnetic resonance (^1^H-NMR) spectroscopy according to the standardized protocol described by Dona et al. (34). Bruker’s *in vitro* diagnostic research (IVDr) procedure was applied. For this, serum samples were thawed and mixed with phosphate buffer (75 mM, pH 7.4) before 600 μL were transferred to the appropriate 5 mm NMR tubes. Two different spectra were collected for each sample: One-dimensional (1D) Nuclear Overhauser Enhancement Spectroscopy (NOESY) spectrum (pulse program: noesygppr1d) and a 1D Carr–Purcell–Meiboom–Gill (CPMG) spin-echo spectrum (pulse program: cpmgpr1d). 39 metabolites and 112 lipoprotein parameters were automatically determined by Bruker Quantification in Plasma/Serum (B.I.Quant-PS 2.0.0) and Bruker IVDr Lipoprotein Subclass Analysis (B.I.-LISA). Various very low-density lipoprotein (VLDL), low-density lipoprotein (LDL), intermediate-density lipoprotein (IDL), and high-density lipoprotein (HDL) parameter in cholesterol (Chol), free cholesterol (FC), phospholipids (PL) triglycerides (TG), and apolipoprotein (Apo) were analyzed.

### Statistics

An unpaired, non-parametric Mann-Whitney test was used to determine the comparability of the patients (PSC and EIM) and HC in terms of sex, age, and BMI. In addition, a comparison of sex, age, and BMI between all three groups (PSC, EIM, and HC) was performed using non-parametric Kruskal-Wallis test. Multivariate partial least square discriminant analysis (PLS-DA) with quantified IVDr data was performed in PLS toolbox (Eigenvector Research, Inc. Wenatchee, Washington). Data were variance-scaled, mean-centered, and orthogonal signal corrected. Venetian blinds were used for cross-validation and calculation of the area under the curve (AUC). Furthermore, a random forest analysis with cross-validation was conducted using the program Python with the TensorFlow framework and scikit-learn package. For feature selection, we employed both the Mean Decrease in Impurity (MDI) and Mean Decrease Accuracy methods. The accuracy and standard deviation (SD) were calculated for both models. For univariate comparisons, unpaired multiple Mann-Whitney test (*p* ≤ 0.05) with a false discovery rate of 1% using the method from Benjamini, Krieger, and Yekutieli were applied. For pyruvic acid the receiver operating characteristic (ROC) curve and the corresponding AUC were calculated.

## Results

Demographic data such as sex, age, and body mass index (BMI) from patients with PSC, patients with EIM, and HC are listed in Table 1.

**TABLE 1:** Demographics of the study cohort, consisting of patients with PSC, patients with EIM, and HC.

Demographics	PSC (*n* = 33)	EIM (*n* = 64)	HC (*n* = 40)	*p*-value
Sex, *n* (%)	f: 10 (30)	f: 37 (58)	f: 18 (45)	PSC vs. EIM: 0.0177*
	m: 23 (70)	m: 27 (42)	m: 22 (55)	PSC vs. HC: 0.2330
				EIM vs. HC: 0.2300
				PSC vs. EIM vs. HC: 0.0352*
Median age, y	47.00	43.50	34.50	PSC vs. EIM: 0.6155
				PSC vs. HC: 0.4614
				EIM vs. HC: 0.2961
				PSC vs. EIM vs. HC: 0.4781
Median BMI kg/m^2^	24.11	24.66	24.51	PSC vs. EIM: 0.6657
				PSC vs. HC: 0.9831
				EIM vs. HC: 0.5812
				PSC vs. EIM vs. HC: 0.8262

Age and BMI are given as the median.

*p < 0.05.

PSC patients were subdivided into patients with and without IBD. Age of initial diagnosis, laboratory parameters gamma-glutamyltransferase (gamma-GT), alanine-transaminase (ALT), AP, bilirubin, model of endstage liver disease (MELD) score (≥5, ≥10, ≥15), Child-Pugh stage of liver cirrhosis (none, CHILD A, CHILD B, CHILD C), histological proof of diagnosis, dominant stenosis, performed liver transplantation, cholestasis, portal hypertension/hepatosplenomegaly, and autoimmune hepatitis (AIH)-PSC overlap syndrome were evaluated to phenotype the PSC patients (Table 2).

**TABLE 2:** Characteristics of PSC-IBD patients and PSC patients without IBD.

Characteristics of PSC patients		PSC-IBD (*n* = 24)	PSC w/o IBD (*n* = 9)
Median age initial diagnosis, y		33.93	35.29
Histological proof of PSC (%)	Yes	11 (45.8)	5 (55.6)
	No	7 (29.2)	3 (33.3)
	Missing Data	6 (25.0)	1 (11.1)
Dominant stenosis (%)	Yes	7 (29.2)	5 (55.6)
	No	15 (62.5)	3 (33.3)
	Missing Data	2 (8.3)	1 (1.1)
Median of endoscopic interventions for dominant stenosis in the last 2°years		4	9.5
Median duration of dominant stenosis, y		0.67	0.37
Median Gamma-GT	U/l	46	101
Gamma-GT (%)	<60°U/l	14 (58.3)	2 (22.2)
	≥60°U/l	10 (41.7)	7 (77.8)
Median ALT	U/l	33	27
ALT (%)	≤50°U/l	15 (62.5)	6 (66.7)
	>50°U/l	9 (37.5)	3 (33.3)
Median AP	U/l	122	173
AP (%)	<130°U/l	13 (54.2)	2 (22.2)
	≥130°U/l	11 (45.8)	7 (77.8)
Median total bilirubin	mg/dl	0.7	1.5
Total bilirubin (%)	≤1.4 mg/dl	19 (97.2)	4 (44.4)
	>1.4 mg/dl	5 (20.8)	5 (55.6)
MELD-Score (%)	≥5	16 (66.7)	4 (44.4)
	≥10	3 (12.5)	2 (22.2)
	≥15	2 (8.3)	1 (11.1)
	Missing data	3 (12.5)	2 (22.2)
Liver transplant at the time of sample collection (%)	Yes	5 (20.8)	2 (22.2)
	No	19 (79.2)	7 (77.8)
Liver cirrhosis at the time of sample collection (%)	CHILD A	5 (20.8)	1 (11.1)
	CHILD B	1 (4.2)	1 (11.1)
	CHILD C	0 (0.0)	0 (0.0)
	None	12 (50.0)	7 (77.8)
	Missing data	1 (4.2)	0 (0.0)
AIH-PSC overlap Syndrome (%)	Yes	2 (8.3)	3 (33.3)
	No	21 (87.5)	6 (66.7)
	Missing data	1 (4.2)	0 (0.0)
Cholestasis (%)	Yes	2 (8.3)	0 (0.0)
	No	21 (87.5)	9 (100.0)
	Missing data	1 (4.2)	0 (0.0)
Portal hypertension/ Splenomegaly (%)	Yes	7 (29.2)	3 (33.3)
	No	16 (66.7)	6 (66.7)
	Missing data	1 (4.2)	0 (0.0)

Diagnosis, current disease status (dominant stenosis defined by percutaneous transhepatic cholangiography (PTC), MRCP and ERCP, endoscopic interventions for dominant stenosis (dilatation, stent, and drainage insertion), laboratory parameters, MELD score, cholestasis defined by imaging (sonography, computer tomography, and MRCP), portal hypertension, and splenomegaly), AIH-PSC overlap syndrome, and liver transplantation were collected.

Table 3 describes EIM and previous gastrointestinal surgeries for patients with PSC-IBD (patients with PSC-IBD as listed in Table 2) and patients with EIM. To get an impression of the current disease activity of IBD, fecal calprotectin, histological remission, and Gastrointestinal Symptom Rating Scale (GSRS) questionnaires with a maximum of 91 points in total and division into 4 groups (no complaints: 13; minor complaints 14-39; moderate complaints 40-65; strong complaints: 66-91) were used (Table 3).

**TABLE 3:** Characteristics of patients with IBD.

Characteristics of IBD patients		PSC-IBD (*n* = 24)	EIM (*n* = 64)
Subgroup (%)	UC	20 (83.3)	13 (20.3)
	Crohn’s Disease (CD)	4 (16.7)	51 (79.7)
GSRS (%)	None	0 (0.0)	3 (4.7)
	Minor complaints	17 (70.8)	37 (57.8)
	Moderate complaints	2 (8.3)	18 (28.1)
	Strong complaints	0 (0.0)	3 (4.7)
	Missing data	5 (20.8)	3 (4.7)
Calprotectin (%)	≤50 μg/g	12 (50.0)	30 (46.9)
	≤150 μg/g	4 (16.7)	22 (34.3)
	>150 μg/g	3 (12.5)	6 (9.4)
	≥500 μg/g	1 (4.2)	5 (7.8)
	Missing data	4 (16.7)	1 (1.6)
Gastrointestinal surgery (%)	Total colectomy	3 (12.5)	4 (6.3)
	Ileocecal resection	2 (8.3)	16 (25.0)
	Appendectomy	2 (8.3)	8 (12.5)
	Gallbladder removal	7 (29.2)	5 (7.8)
	Fistula surgery	1 (4.2)	12 (18.8)
	Other	9 (37.5)	18 (28.1)
	None	8 (33.3)	28 (43.8)
Other extraintestinal manifestations (%)	Arthralgia	6 (25)	47 (73.4)
	Skin involvement	1 (4.2)	24 (37.5)
	Eye involvement	0 (0.0)	18 (28.1)

Comparison of PSC-IBD patients and EIM patients without PSC. The phenotype of the disease, current disease activity (GSRS and calprotectin) gastrointestinal surgeries, and other extraintestinal manifestations were collected.

### Patients with PSC, EIM, and HC display distinct metabolome and lipidome profiles

NMR spectroscopy was used to distinguish metabolome and lipidome profiles in patients with PSC, EIM, as well as HC.

**FIGURE 1:**
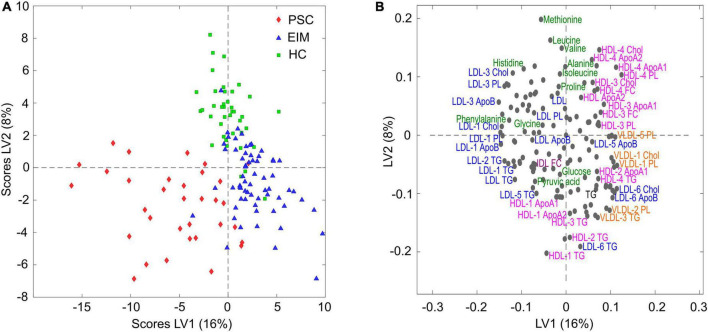
**(A)** Scores plot of PLS-DA for quantified NMR data in patients with PSC (red, *n* = 33), EIM (blue, *n* = 64), and HC (green, *n* = 40). **(B)** Corresponding loadings plot of distinct metabolome and lipidome profiles. Axis label: latent variable (LV).

Scores plot of PLS-DA for quantified IVDr data shows a separation of patients with PSC (red), patients with EIM (blue), and HC (green) (Figure 1A). Corresponding metabolites and lipoproteins that are decisive for good discrimination are shown in the loadings plot (Figure 1B). Amino acids and several components of LDL, and HDL subfractions differed between the groups. The AUC values for cross-validation of 0.87 for PSC, 0.84 for HC, and 0.86 for EIM indicate a suitable model for the discrimination of PSC, EIM, and HC (Supplementary Figure 1). Additionally, we conducted a random forest analysis with cross-validation. On one hand, the model using Mean Decrease in Impurity (MDI) yielded an accuracy of 89.6% (SD: 0.054), and on the other hand, Mean Decrease Accuracy resulted in 79.3% (SD: 0.095). The parameters contributing to the discrimination between the three groups in both models, achieving a high score (MDI > 0.01 and Mean Decrease in Accuracy > 0), are presented in Supplementary Figure 2.

### Analysis of patients with PSC compared with HC and patients with EIM revealed significant differences

**FIGURE 2:**
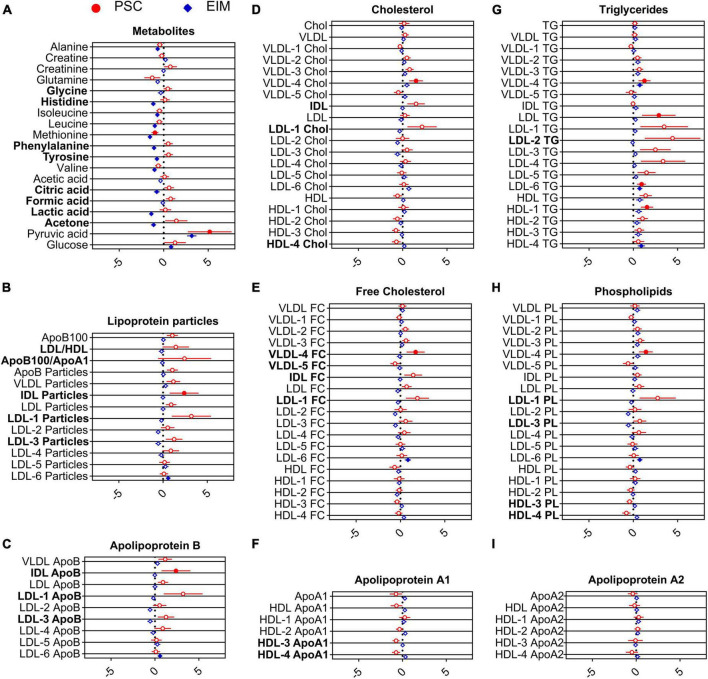
Adjusted forest plot demonstrate the differences of metabolites **(A)** and lipoproteins **(B–I)** from PSC (red, *n = 33*) and EIM (blue, *n = 64*) compared to HC normalized to the standard deviation of HC. The dashed vertical line indicates the reference values for HC. Statistically significant values of PSC and EIM compared with HC are shown by color-filled circles and squares, and parameters that significantly differed between PSC and EIM are written in bold letters.

The adjusted forest plots in Figure 2 display the differences between PSC (red) and EIM (blue) compared to the HC, respectively, and normalized to the standard deviation of HC in metabolites and lipoproteins. The dashed vertical line serves as the mean reference range of the HC, while the horizontal dots indicate the deviation from the reference. The color-filled shapes demonstrate statistically significant parameters, while color-filled red circles indicate the significance of PSC to HC, and blue color-filled squares indicate the significance of EIM to HC. Bold printed names of metabolites and lipoproteins indicate statistical significance between PSC and EIM (Supplementary Table 1).

Pyruvic acid concentrations were markedly enhanced in PSC compared to HC (Figure 2A), while the level of methionine was diminished in PSC compared to HC (Figure 2A).

Decreased levels of alanine, histidine, isoleucine, leucine, methionine, phenylalanine, tyrosine, valine, lactic acid, citric acid, and acetone in EIM compared with those in HC were observed (Figure 2A). Conversely, pyruvic acid and glucose levels were higher in EIM (Figure 2A) than in HC.

When comparing metabolites from patients with PSC with the EIM group, elevated serum concentrations of glycine, histidine, phenylalanine, tyrosine, citric acid, formic acid, lactic acid, and acetone were detected (Figure 2A).

Taken together, the most notable metabolic alteration in PSC was the significant increase in pyruvic acid concentration compared to HC (Figure 2A).

### Lipidomic shifts distinguish patients with PSC compared with HC and patients with EIM

Figures 2B–I is additionally demonstrate serum concentrations of lipoprotein parameters. In particular, VLDL-4 lipids, including Chol levels, FC concentrations, TG, and PL, were significantly increased in PSC compared with HC. Moreover, elevated concentrations of LDL TG, LDL-6 TG, and HDL-1 TG were noted (Figure 2G). Significantly increased levels of IDL particles (Figure 2B), and accordingly, IDL ApoB (Figure 2C) were observed in PSC.

The lipidomic profile of patients with EIM differed from that of patients with HC by increased levels of LDL-6 particles as well as higher levels of ApoB, FC, TG, and PL (Figures 2B–H). VLDL-4 and HDL-4 showed increased TG levels (Figure 2G).

The ratio of LDL and HDL, the ratio of ApoB100 and ApoA1, and several subfractions of HDL (HDL-3 ApoA1, HDL-4 ApoA1, HDL-3 PL, and HDL-4 PL) were decreased in PSC compared with EIM (Figures 2B–H). Higher concentrations of LDL-1 particles, ApoB, Chol, FC, and PL and LDL-3 particles, ApoB, PL, and LDL-2 TG were detected in PSC compared with those in the EIM group (Figures 2B, E, G, H). In comparison to EIM, increased concentrations of IDL particles, Chol, and FC were observed (Figures 2B, D, E). In PSC, VLDL-4 showed higher FC levels and VLDL-5 showed lower FC levels than in EIM.

Notably, the concentrations of IDL-ApoB, IDL particles, and VLDL-4 were significantly altered in PSC compared with both, EIM and HC. These results contribute to a distinction of the PSC patients based on the lipidome profile.

**FIGURE 3:**
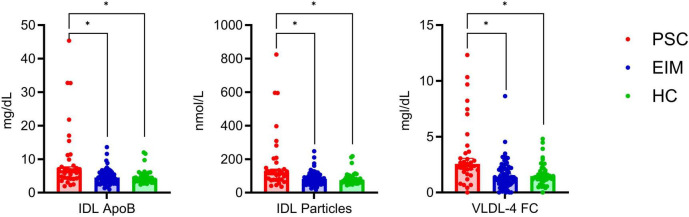
Detailed comparison of altered lipoprotein concentrations in PSC (red, *n* = 33), EIM (blue, *n* = 64), and HC (green, *n* = 40) given as median with 95% confidence interval. The levels of IDL ApoB, IDL particles, and VLDL-4 FC significantly differed between PSC and EIM as well as between PSC and HC. Significant differences determined using the unpaired multiple Mann-Whitney test (*p* < 0.05) with a false discovery rate of 1% using the method from Benjamini were highlighted (*).

In Figure 3, these lipoproteins with altered concentrations are depicted. Levels of IDL particles in patients with PSC significantly differed from EIM and HC. A strong increase in the concentration of ApoB in IDL could be observed in PSC in comparison with EIM but also in comparison with HC. Patients with PSC also displayed elevated FC concentrations in VLDL-4 subfraction.

### Specific metabolome and lipidome profile of patients with PSC compared to HC

**FIGURE 4:**
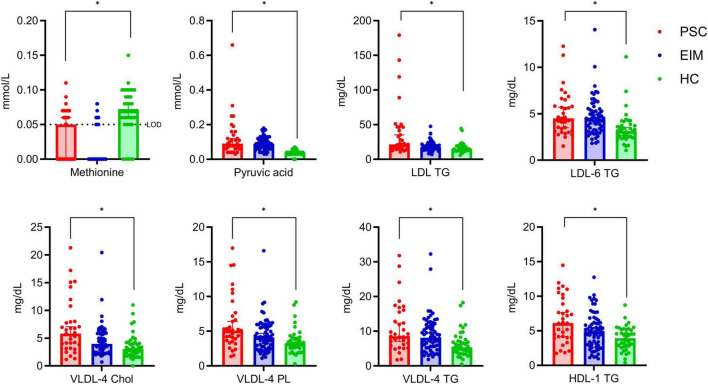
Detailed comparison of significant altered metabolites and lipoprotein subfractions between PSC (red, *n* = 33) and HC (green, *n* = 40), but not in comparison with EIM (blue, *n* = 64). Concentrations are given as median with 95% confidence interval. The lower limit of detection (LOD) for methionine is 0.05 mmol/L. Significant differences determined using the unpaired multiple Mann-Whitney test (*p* < 0.05) with a false discovery rate of 1% using the method from Benjamini were highlighted (*).

To summarize the significant changes in PSC compared with HC from Figure 2 in detail, except of changes already mentioned in Figure 3, the relevant metabolites and lipoproteins are displayed in Figure 4.

Altered metabolites include lower levels of methionine, and higher levels of pyruvic acid in PSC in comparison to HC (Figure 4). VLDL-4 bound lipids in Chol, PL, and TG are increased in PSC, as well as concentrations of LDL TG, LDL-6 TG, and HDL-1 TG (Figure 4). These results are specific to the alterations in PSC compared with HC in our study since none of the lipoproteins and metabolites in Figure 4 showed a significant change between PSC and EIM.

**FIGURE 5:**
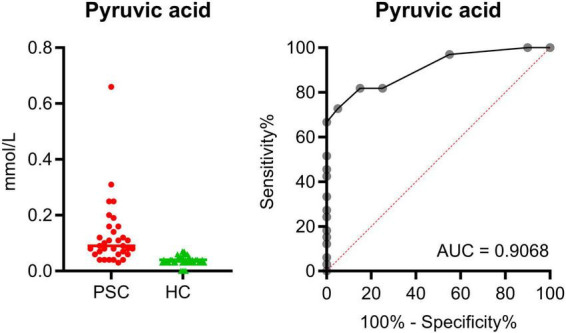
Total concentration of pyruvic acid in comparison of PSC (red, *n* = 33) and HC (green, *n* = 40). Display of the ROC with an AUC of 0.9068.

Pyruvic acid, the metabolite with the strongest differentiation in comparison between PSC and HC, is displayed separately with total concentrations (Figure 5). Figure 5 illustrates the ROC curve with corresponding AUC of 0.9068, which presents a good separation between the groups. The best Youden Index was calculated with 67.73 using a cut-off of 0.065 mmol/L pyruvic acid (Supplementary Table 2).

## Discussion

To advance the diagnosis of PSC our study utilizes serum metabolome and lipidome signatures to complement imaging techniques. Currently, the diagnosis of PSC is mainly based on image morphology, either by MRCP or by ERCP. However, due to the lack of PSC-specific biomarkers, highly sensitive diagnosis remains challenging. Our study aims to contribute to the diagnosis of PSC based on changes in the serum metabolome and lipidome to enable early diagnosis by fingerprints alongside the use of imaging techniques.

In about 70% of cases, PSC is associated with IBD (4). However, there is evidence that PSC is constantly associated with inflammation in the colon, which in some patients can only be mapped at the molecular level (35). This leads to the conclusion, that PSC can mostly be considered as a serve EIM of IBD. In addition, several studies have shown that the clinical phenotype of PSC-IBD patients differs from IBD patients without PSC (36, 37). For this reason, all PSC patients (*n* = 33) were included in one group, even those who did not have clinically confirmed IBD and we compared them with both HC and EIM patients including eye, skin, and joint involvement.

To the best of our knowledge, there is no NMR spectroscopic analysis that specifically addresses the distinction between PSC-IBD and IBD without PSC but other EIM by analyzing the serum metabolome and lipidome.

The main results of our serum metabolome comparisons of PSC and HC reveal a decrease in methionine and an increase in pyruvic acid.

Methionine is involved in various metabolic processes, including fat metabolism, endogenous antioxidation, and protein biosynthesis (38, 39). A decrease in methionine concentration, as observed in our study, may lead to an impairment of these important metabolic processes.

Inflammation increases aerobic glycolysis (40), resulting in higher levels of pyruvate. Increased pyruvic acid concentration was found in both PSC and EIM patients compared to HC, probably related to the higher glycolysis triggered by chronic inflammation.

Contrary to our study, lower levels of branched-chain amino acids (BBAAs) were found in plasma of patients with PSC (29, 41). However, our study could not confirm these results. There are several explanations for this such as dietary bias (see limitations).

Furthermore, we detected changes in serum concentrations of acetone, various amino acids (histidine, phenylalanine, tyrosine, and glycine), and organic acids (citric acid, lactic acid, and formic acid).

Increased concentrations of the ketone bodies 3-hydroxybutyrate and acetoacetate in patients with PSC compared to HC have been described by Bell et al. (11). In contrast, no significant change in the concentration of ketone bodies was found in PSC compared to HC in this study and in the study of Radford-Smith et al. (30). Of relevance, increased acetone levels of patients with PSC compared to EIM were noted.

In addition, we describe higher histidine concentrations in patients with PSC compared to patients with EIM. A low concentration of histidine in the plasma of patients with UC is related to a higher risk of acute relapse (42, 43). These results support the hypothesis that there is no dependence between IBD activity and PSC progression (44).

In patients with advanced liver cirrhosis, progressively elevated levels of aromatic amino acids (AAA) are observed (45, 46). Since six of eight PSC patients with cirrhosis were found to have only a mild form of cirrhosis (Table 2), a significant increase in AAA compared to HC was not expected. The study by ter Borg et al. (41) also noted no significant changes in AAA in PSC compared to HC, but trends toward higher phenylalanine and tyrosine levels, and trends toward reduced tryptophan levels. Considering the comparison of PSC and EIM, increased concentration of tyrosine and phenylalanine in PSC were noted. Lower levels of tyrosine in IBD patients compared to HC are previously described by Schicho et al. (47).

In our study, increased levels of citric acid in PSC patients compared to EIM were detected. We also noted changes between EIM and HC, which are consistent with the lower citrate levels in IBD patients compared to HC determined by Schicho et al. (47).

The concentrations of formic acid, lactic acid, and glycine were higher in PSC patients compared to patients with EIM. However, in previous studies, increased concentrations of these metabolites were noted in IBD patients compared to HC (47, 48).

Considering the lipidome profile of patients with PSC compared to HC as well as EIM, increased concentrations of IDL particles, IDL ApoB, and VLDL-4 FC were demonstrated. In addition, VLDL-4 bound lipids in Chol, TG, and PL were higher in PSC than in HC. Considering the changes in PSC compared to EIM, several LDL subclasses displayed an increase in ApoB, Chol, FC, TG, and PL. Furthermore, PSC patients showed increased LDL to HDL ratios, and accordingly a higher ApoB100 to ApoA1 ratio compared with EIM, which may present an atherogenic lipid profile of PSC patients (49, 50).

High concentrations of free fatty acids induce the formation of VLDL by enhancing hepatic triglyceride synthesis (51, 52), which in turn is metabolized to IDL particles (53). Thus, the results of Bell et al. (11), demonstrating increases in free fatty acids (linoleate, linolenate, palmitoleate, oleate) in the serum, are in agreement with the increased levels of IDL particles. Increased numbers of IDL particles appear to be atherogenic (54). Similarly, plasma samples of patients with non-insulin-dependent diabetes displayed elevated levels of IDL ApoB, which are potentially atherogenic (55). Concentrations of Chol, TG, ApoB, and ApoA1, are associated with coronary heart disease and carotid plaques (56). Large VLDL particles found in insulin-resistant patients with diabetes mellitus type 2 are associated with a higher risk of developing cardiovascular disease (57). In our study, the components of VLDL-4 particles were elevated. Notably, an association between small VLDL and atherosclerotic risk was reported before (58).

To sum up the distinct metabolome and lipidome profiles of PSC patients compared to IBD patients with other EIM detected by our study corroborate the importance of considering IBD with and without PSC as individual entities.

Furthermore, we studied the impact of the different therapies (UDCA, mesalazine, and biologicals) on the metabolome and lipidome of PSC patients. We did not observe significant changes, but trends to higher LDL-1 to LDL-3 TG levels under UDCA therapy compared to therapy with UDCA and biologicals (see Supplementary Figure 3 and Table 3). The attribution of metabolomic and lipidomic changes to a specific drug is generally difficult because of the inclusion of additional medication alongside UDCA, mesalazine, and biologicals. For a clear differentiation of the metabolome and lipidome among the specific therapies, a larger number of subjects will be required in the future.

We hypothesize that the specific metabolomic profile of PSC patients (Figures 1A, 2B) contribute to the promotion of a pro-inflammatory milieu in the intrahepatic cholangiocyte and portal triad (23–25). The proinflammatory milieu is the basis of structural damage to the bile ducts with stenosis and progressive liver parenchymal damage (59, 60). Genetic predisposition, epigenetic changes and also metabolomic changes are prerequisites for disease development and progression in PSC (60, 61).

In addition to the restricted number of patients, a further limitation of our study is that strict adherence to fasting state before blood sample collection in patients and HC could not always be guaranteed. This leads to a potential dietary bias in the data. Of note, we obtained clear results based on detailed clinical phenotyping, comparison with sex-, age-, and BMI-matched HC, and first-time comparison with EIM using NMR spectroscopy. The recruitment of sufficient study participants in a rare cohort as PSC is generally difficult. This is precisely why the significant differences revealed by our study are of particular clinical relevance. For this reason, there should be further research focusing on the distinct metabolome and lipidome of PSC patients.

## Conclusions

A specific serum metabolome and lipidome profile was detected in PSC patients and it differs from patients with EIM and HC. So far, the diagnosis of PSC is mainly established based on various imaging techniques (12). Our data suggest that the detection of PSC can be improved by using NMR spectroscopy. In conclusion, our research enhances PSC diagnosis by integrating serum metabolome and lipidome signatures with traditional imaging techniques.

## Data availability statement

The raw data supporting the conclusions of this article are provided by the authors without undue reservation.

## Ethics statement

The studies involving humans were approved by the Ethics Committee of the University of Regensburg and the University of Lübeck (No. 21-2390-101 and No. 22-104). The studies were conducted in accordance with the local legislation and institutional requirements. The participants provided their written informed consent to participate in this study.

## Author contributions

TF: Conceptualization, Data curation, Investigation, Resources, Validation, Writing – original draft. CB: Conceptualization, Project administration, Resources, Supervision, Writing – review & editing, Visualization. AK: Conceptualization, Resources, Writing – review & editing. TE: Data curation, Project administration, Resources, Writing – review & editing. JL: Data curation, Resources, Writing – review & editing. SSc: Writing – review & editing. SSo: Data curation, Resources, Writing – review & editing. SG: Data curation, Resources, Writing – review & editing. SZ: Writing – review & editing. MH: Data curation, Writing – review & editing. DB: Writing – review & editing. SK: Writing – review & editing. SW: Writing – review & editing. BF: Writing – review & editing. XH: Formal analysis, Writing – review & editing. MG: Formal analysis, Writing – review & editing. S-DP: Writing – review & editing. UG: Software, Validation, Writing – review & editing. JM: Writing – review & editing. CK: Writing – review & editing. KG: Writing – review & editing. MM: Conceptualization, Project administration, Supervision, Writing – review & editing. CS: Conceptualization, Methodology, Project administration, Supervision, Writing – review & editing. FS: Conceptualization, Formal analysis, Investigation, Methodology, Project administration, Software, Supervision, Validation, Visualization, Writing – review & editing. HT: Conceptualization, Investigation, Methodology, Project administration, Resources, Supervision, Validation, Writing – review & editing.
