# Supplementary material for: Distinct metabolomic and lipidomic profiles in serum samples of patients with primary sclerosing cholangitis
Source: Front Med (Lausanne). 2024 Jun 4;11:1334865. doi: 10.3389/fmed.2024.1334865 (PMC11184724; doi:10.3389/fmed.2024.1334865)
Supplement: Supplementary file 1 [file Data_Sheet_1.docx]

Distinct metabolomic and lipidomic profiles in serum samples of patients with primary sclerosing cholangitis

Tanja Fererberger^1^, Christa Buechler^1^, Arne Kandulski^1^, Tanja Elger^1^, Johanna Loibl^1^, Stephan Schmid^1^, Stefanie Sommersberger^1^, Stefan Gunawan^1^, Sebastian Zundler^6,7^, Muriel Huss^1^, Dominik Bettenworth^8,9^, Sally Kempa^10^, Simon Weidlich^11^, Bandik Föh^2,3^, Xinyu Huang^4^, Marcin Grzegorzek^4^, Stefanie Derer-Petersen^2^, Ulrich L. Günther^5^, Jens U. Marquardt^3^, Claudia Kunst^1^, Karsten Gülow^1^, Martina Müller^1^, Christian Sina^2,3,12^, Franziska Schmelter^2†^, Hauke C. Tews^1†^

^†^These authors share senior authorship

^1^ Department of Internal Medicine I, Gastroenterology, Hepatology, Endocrinology, Rheumatology, and Infectious diseases, University Hospital Regensburg, Regensburg, Germany

^2^ Institute of Nutritional Medicine, University Medical Center Schleswig-Holstein, Campus Lübeck, Lübeck, Germany

^3^ Department of Medicine I, University Medical Center Schleswig-Holstein, Campus Lübeck, Lübeck, Germany

^4^ Institute of Medical Informatics, University of Lübeck, Germany

^5^ Institute of Chemistry and Metabolomics, University of Lübeck, Lübeck, Germany

^6^ Department of Medicine 1, University Hospital Erlangen, Friedrich-Alexander-Universität Erlangen-Nürnberg, Erlangen-Nürnberg, Germany.

^7^ Deutsches Zentrum Immuntherapie, University Hospital Erlangen, Erlangen, Germany

^8^ University Hospital Münster, Department of Medicine B - Gastroenterology and Hepatology, Münster, Germany

^9^ Practice for Internal Medicine, Münster, Germany

^10^ Department for Plastic, Hand, and Reconstructive Surgery, University Hospital Regensburg, Regensburg, Germany

^11^ Technical University of Munich, School of Medicine and Health, University Hospital Rechts der Isar, Department of Internal Medicine II, Munich, Germany

^12^ Fraunhofer Research Institution for Individualized and Cell-Based Medical Engineering (IMTE), Lübeck, Germany

*** Correspondence:**Hauke Christian Tews, hauke.tews@ukr.de

**Supplementary Table S1: P-, and q-values of metabolites and lipoproteins for comparison of patients and healthy controls.** Quantified metabolites and lipoproteins analyzed using nuclear magnetic resonance (NMR) spectroscopy with p- and q-values (unpaired Mann-Whitney test with false discovery rate of 1% using the correction from Benjamini, Krieger, Yekutieli) for the comparison of PSC patients and EIM, PSC and healthy controls (HC) as well as EIM and HC.

|  | **PSC vs EIM** |  | **PSC vs HC** |  | **EIM vs HC** |  |
| --- | --- | --- | --- | --- | --- | --- |
|  | p-value | q-value | p-value | q-value | p-value | q-value |
| Ethanol | 0.463442 | 0.448336 | 0.452055 | 0.552495 | 0.158057 | 0.30033 |
| Trimethylamine-N-oxide | 0.0622 | 0.097811 | 0.221815 | 0.329648 | >0.999999 | 0.866645 |
| 2-Aminobutyric acid | 0.005496 | 0.017802 | 0.697105 | 0.756619 | 0.000694 | 0.005457 |
| Alanine | 0.178935 | 0.226828 | 0.084059 | 0.165209 | 0.000605 | 0.005415 |
| Asparagine | 0.000008 | 0.00011 | 0.096653 | 0.182562 | 0.000985 | 0.006618 |
| Creatine | 0.04777 | 0.083583 | 0.450795 | 0.552495 | 0.355868 | 0.510044 |
| Creatinine | 0.121674 | 0.169838 | 0.138044 | 0.244843 | 0.947684 | 0.866645 |
| Glutamic acid | <0.000001 | <0.000001 | 0.001135 | 0.012496 | <0.000001 | 0.000002 |
| Glutamine | 0.472106 | 0.451151 | 0.013924 | 0.049392 | 0.003085 | 0.015348 |
| Glycine | 0.000835 | 0.004509 | 0.062317 | 0.137325 | 0.036291 | 0.0975 |
| Histidine | 0.000006 | 0.000105 | 0.988832 | 0.938323 | <0.000001 | 0.000001 |
| Isoleucine | 0.270597 | 0.305603 | 0.058829 | 0.131631 | 0.000152 | 0.001566 |
| Leucine | 0.018492 | 0.03766 | 0.058453 | 0.131631 | <0.000001 | 0.000002 |
| Lysine | 0.310405 | 0.336207 | 0.006 | 0.033864 | 0.004987 | 0.021608 |
| Methionine | 0.006292 | 0.018177 | 0.000613 | 0.008856 | <0.000001 | <0.000001 |
| N.N-Dimethylglycine | >0.999999 | 0.801484 | 0.200913 | 0.31086 | 0.158057 | 0.30033 |
| Ornithine | 0.007254 | 0.01959 | 0.971333 | 0.938323 | 0.000969 | 0.006618 |
| Phenylalanine | <0.000001 | <0.000001 | 0.158154 | 0.264389 | <0.000001 | <0.000001 |
| Proline | 0.414621 | 0.415391 | 0.075139 | 0.151781 | 0.004335 | 0.019939 |
| Sarcosine | 0.486158 | 0.457541 | 0.567696 | 0.635121 | 0.978226 | 0.866645 |
| Threonine | 0.000586 | 0.003624 | 0.14228 | 0.246347 | 0.013743 | 0.048581 |
| Tyrosine | 0.000005 | 0.000105 | 0.172929 | 0.279454 | 0.000286 | 0.002741 |
| Valine | 0.016895 | 0.035341 | 0.054015 | 0.126708 | <0.000001 | 0.000005 |
| 2-Hydroxybutyric acid | 0.395985 | 0.406555 | 0.200913 | 0.31086 | >0.999999 | 0.866645 |
| Acetic acid | 0.153857 | 0.207336 | 0.954511 | 0.937522 | 0.155464 | 0.30033 |
| Citric acid | 0.000008 | 0.00011 | 0.048024 | 0.114502 | 0.001663 | 0.009154 |
| Formic acid | 0.001264 | 0.005608 | 0.013179 | 0.047917 | 0.54661 | 0.606828 |
| Lactic acid | <0.000001 | 0.000012 | 0.734638 | 0.78005 | <0.000001 | <0.000001 |
| Succinic acid | 0.005838 | 0.017802 | 0.002622 | 0.021185 | 0.522031 | 0.601229 |
| Choline | 0.037005 | 0.066625 | 0.087723 | 0.170112 | >0.999999 | 0.866645 |
| 2-Oxoglutaric acid | 0.003683 | 0.013072 | 0.015801 | 0.053444 | >0.999999 | 0.866645 |
| 3-Hydroxybutyric acid | 0.000456 | 0.002982 | 0.040862 | 0.108053 | 0.009317 | 0.033824 |
| Acetoacetic acid | 0.050141 | 0.085329 | 0.021854 | 0.068881 | 0.512478 | 0.601229 |
| Acetone | 0.000002 | 0.000059 | 0.20473 | 0.31343 | <0.000001 | 0.000001 |
| Pyruvic acid | 0.509251 | 0.475671 | <0.000001 | <0.000001 | <0.000001 | <0.000001 |
| D-Galactose | >0.999999 | 0.801484 | >0.999999 | 0.938323 | 0.384615 | 0.523149 |
| Glucose | 0.479375 | 0.454601 | 0.012859 | 0.047917 | 0.000017 | 0.000205 |
| Glycerol | 0.032096 | 0.060413 | 0.112163 | 0.203913 | 0.000007 | 0.000096 |
| Dimethylsulfone | 0.913382 | 0.766685 | 0.146393 | 0.250488 | 0.089137 | 0.199562 |
| TG | 0.663113 | 0.592652 | 0.351137 | 0.482522 | 0.520766 | 0.601229 |
| Chol | 0.824544 | 0.701597 | 0.821346 | 0.853261 | 0.512151 | 0.601229 |
| LDL | 0.405293 | 0.409346 | 0.879618 | 0.888414 | 0.434124 | 0.566174 |
| HDL | 0.028485 | 0.054442 | 0.042605 | 0.108709 | 0.740713 | 0.726278 |
| ApoA1 | 0.01377 | 0.031103 | 0.225327 | 0.331025 | 0.158739 | 0.30033 |
| ApoA2 | 0.229451 | 0.274083 | 0.35256 | 0.482522 | 0.705808 | 0.712866 |
| ApoB100 | 0.00843 | 0.021817 | 0.011827 | 0.047782 | 0.998616 | 0.866645 |
| LDL/HDL | 0.001806 | 0.007465 | 0.022259 | 0.068881 | 0.323392 | 0.49365 |
| ApoB100/ApoA1 | 0.001863 | 0.007465 | 0.010929 | 0.046749 | 0.611754 | 0.652198 |
| ApoB Particles | 0.00843 | 0.021817 | 0.011827 | 0.047782 | >0.999999 | 0.866645 |
| VLDL Particles | 0.156823 | 0.207336 | 0.013034 | 0.047917 | 0.144865 | 0.294843 |
| IDL Particles | 0.000401 | 0.00277 | 0.000532 | 0.008856 | 0.945654 | 0.866645 |
| LDL Particles | 0.01239 | 0.029046 | 0.038189 | 0.102855 | 0.702106 | 0.712866 |
| LDL-1 Particles | 0.000111 | 0.001134 | 0.005205 | 0.033864 | 0.247587 | 0.416283 |
| LDL-2 Particles | 0.012119 | 0.029046 | 0.430385 | 0.54908 | 0.008835 | 0.032967 |
| LDL-3 Particles | 0.000136 | 0.001134 | 0.045893 | 0.11313 | 0.016992 | 0.054818 |
| LDL-4 Particles | 0.086976 | 0.128632 | 0.327341 | 0.461615 | 0.308882 | 0.479331 |
| LDL-5 Particles | 0.692202 | 0.612339 | 0.945104 | 0.935075 | 0.458977 | 0.586822 |
| LDL-6 Particles | 0.11269 | 0.159085 | 0.404957 | 0.527792 | 0.001704 | 0.009154 |
| VLDL TG | 0.839574 | 0.709526 | 0.548025 | 0.617866 | 0.333361 | 0.50315 |
| IDL TG | 0.365666 | 0.381737 | 0.96047 | 0.937522 | 0.290248 | 0.458694 |
| LDL TG | 0.006704 | 0.018928 | 0.00019 | 0.0083 | 0.040353 | 0.103116 |
| HDL TG | 0.137655 | 0.187921 | 0.001214 | 0.012496 | 0.039697 | 0.103116 |
| VLDL | 0.531812 | 0.493037 | 0.243899 | 0.352828 | 0.495145 | 0.601229 |
| IDL | 0.00081 | 0.004509 | 0.001431 | 0.013005 | 0.990714 | 0.866645 |
| LDL | 0.405293 | 0.409346 | 0.879618 | 0.888414 | 0.434124 | 0.566174 |
| HDL | 0.028485 | 0.054442 | 0.042605 | 0.108709 | 0.740713 | 0.726278 |
| VLDL FC | 0.792388 | 0.683599 | 0.445066 | 0.552495 | 0.242822 | 0.416283 |
| IDL FC | 0.002013 | 0.007816 | 0.006962 | 0.036162 | 0.908666 | 0.866645 |
| LDL FC | 0.005498 | 0.017802 | 0.073323 | 0.150199 | 0.242156 | 0.416283 |
| HDL FC | 0.181773 | 0.228098 | 0.047425 | 0.114502 | 0.248262 | 0.416283 |
| VLDL PL | 0.315588 | 0.336207 | 0.79786 | 0.834825 | 0.086303 | 0.196492 |
| IDL PL | 0.232487 | 0.275065 | 0.071092 | 0.147708 | 0.22319 | 0.394489 |
| LDL PL | 0.048891 | 0.084358 | 0.099406 | 0.185354 | 0.730683 | 0.726278 |
| HDL PL | 0.023907 | 0.047142 | 0.271909 | 0.387711 | 0.172588 | 0.317586 |
| HDL ApoA1 | 0.017069 | 0.035341 | 0.222122 | 0.329648 | 0.131925 | 0.281294 |
| HDL ApoA2 | 0.43821 | 0.435511 | 0.594898 | 0.65547 | 0.794051 | 0.767373 |
| VLDL ApoB | 0.156883 | 0.207336 | 0.012931 | 0.047917 | 0.142559 | 0.294615 |
| IDL ApoB | 0.000389 | 0.00277 | 0.000527 | 0.008856 | 0.94036 | 0.866645 |
| LDL ApoB | 0.012597 | 0.029046 | 0.038189 | 0.102855 | 0.702106 | 0.712866 |
| VLDL-1 TG | 0.223581 | 0.269665 | 0.152007 | 0.257069 | 0.923178 | 0.866645 |
| VLDL-2 TG | 0.776377 | 0.67447 | 0.090547 | 0.173279 | 0.018797 | 0.057386 |
| VLDL-3 TG | 0.605054 | 0.548656 | 0.009075 | 0.042576 | 0.004453 | 0.019939 |
| VLDL-4 TG | 0.276113 | 0.309023 | 0.000342 | 0.0083 | 0.001347 | 0.008228 |
| VLDL-5 TG | 0.053969 | 0.089394 | 0.065134 | 0.141389 | 0.595681 | 0.645305 |
| VLDL-1 Chol | 0.266954 | 0.305603 | 0.141867 | 0.246347 | 0.450959 | 0.582475 |
| VLDL-2 Chol | 0.451785 | 0.441931 | 0.083285 | 0.165209 | 0.093289 | 0.205435 |
| VLDL-3 Chol | 0.071304 | 0.108026 | 0.002012 | 0.01721 | 0.040684 | 0.103116 |
| VLDL-4 Chol | 0.015001 | 0.032694 | 0.000272 | 0.0083 | 0.014338 | 0.049386 |
| VLDL-5 Chol | 0.002327 | 0.008502 | 0.01536 | 0.053188 | 0.385788 | 0.523149 |
| VLDL-1 FC | 0.198523 | 0.246625 | 0.406441 | 0.527792 | 0.528351 | 0.601229 |
| VLDL-2 FC | 0.051606 | 0.086634 | 0.026578 | 0.078479 | 0.609597 | 0.652198 |
| VLDL-3 FC | 0.04671 | 0.082897 | 0.006381 | 0.034373 | 0.17205 | 0.317586 |
| VLDL-4 FC | 0.000132 | 0.001134 | 0.00067 | 0.008856 | 0.489847 | 0.601229 |
| VLDL-5 FC | 0.012626 | 0.029046 | 0.001289 | 0.012496 | 0.50231 | 0.601229 |
| VLDL-1 PL | 0.133596 | 0.184406 | 0.208671 | 0.316136 | 0.637869 | 0.664225 |
| VLDL-2 PL | 0.61302 | 0.551851 | 0.171196 | 0.279454 | 0.008024 | 0.031701 |
| VLDL-3 PL | 0.388329 | 0.402018 | 0.00855 | 0.041453 | 0.015609 | 0.052418 |
| VLDL-4 PL | 0.060539 | 0.097673 | 0.00023 | 0.0083 | 0.02193 | 0.064614 |
| VLDL-5 PL | 0.003953 | 0.013643 | 0.010421 | 0.045927 | 0.502533 | 0.601229 |
| LDL-1 TG | 0.023306 | 0.046699 | 0.005443 | 0.033864 | 0.265091 | 0.429032 |
| LDL-2 TG | 0.000159 | 0.001234 | 0.001048 | 0.012496 | 0.628378 | 0.664225 |
| LDL-3 TG | 0.06001 | 0.097673 | 0.019132 | 0.061835 | 0.255153 | 0.417984 |
| LDL-4 TG | 0.01484 | 0.032694 | 0.005947 | 0.033864 | 0.433132 | 0.566174 |
| LDL-5 TG | 0.166031 | 0.21567 | 0.02698 | 0.078479 | 0.051353 | 0.123183 |
| LDL-6 TG | 0.563375 | 0.518431 | 0.000327 | 0.0083 | 0.000072 | 0.000801 |
| LDL-1 Chol | 0.001081 | 0.004973 | 0.069785 | 0.147095 | 0.06093 | 0.143591 |
| LDL-2 Chol | 0.270267 | 0.305603 | 0.718156 | 0.773693 | 0.006802 | 0.028281 |
| LDL-3 Chol | 0.005947 | 0.017802 | 0.419164 | 0.539498 | 0.017959 | 0.056104 |
| LDL-4 Chol | 0.317236 | 0.336207 | 0.734783 | 0.78005 | 0.355345 | 0.510044 |
| LDL-5 Chol | 0.311878 | 0.336207 | 0.47857 | 0.574751 | 0.713241 | 0.714998 |
| LDL-6 Chol | 0.092137 | 0.133096 | 0.397128 | 0.527792 | 0.002246 | 0.011602 |
| LDL-1 FC | 0.000613 | 0.003624 | 0.032979 | 0.094049 | 0.138039 | 0.289732 |
| LDL-2 FC | 0.246192 | 0.288532 | 0.594898 | 0.65547 | 0.004404 | 0.019939 |
| LDL-3 FC | 0.016347 | 0.035014 | 0.531594 | 0.608128 | 0.022126 | 0.064614 |
| LDL-4 FC | 0.167171 | 0.21567 | 0.503015 | 0.594784 | 0.348582 | 0.510044 |
| LDL-5 FC | 0.465551 | 0.448336 | 0.901365 | 0.9041 | 0.530532 | 0.601229 |
| LDL-6 FC | 0.090655 | 0.132494 | 0.358309 | 0.482522 | 0.000731 | 0.005457 |
| LDL-1 PL | 0.001037 | 0.004953 | 0.024343 | 0.073759 | 0.208295 | 0.373071 |
| LDL-2 PL | 0.068923 | 0.105707 | 0.986819 | 0.938323 | 0.006948 | 0.028281 |
| LDL-3 PL | 0.002153 | 0.008104 | 0.185568 | 0.293359 | 0.023979 | 0.068533 |
| LDL-4 PL | 0.222116 | 0.269665 | 0.482121 | 0.574751 | 0.532753 | 0.601229 |
| LDL-5 PL | 0.295885 | 0.328194 | 0.520665 | 0.605805 | 0.635497 | 0.664225 |
| LDL-6 PL | 0.062142 | 0.097811 | 0.527947 | 0.608128 | 0.001054 | 0.006743 |
| LDL-1 ApoB | 0.000112 | 0.001134 | 0.004976 | 0.033864 | 0.251016 | 0.416283 |
| LDL-2 ApoB | 0.012321 | 0.029046 | 0.43524 | 0.550446 | 0.00879 | 0.032967 |
| LDL-3 ApoB | 0.000137 | 0.001134 | 0.045879 | 0.11313 | 0.017139 | 0.054818 |
| LDL-4 ApoB | 0.085529 | 0.128015 | 0.330088 | 0.461615 | 0.310443 | 0.479331 |
| LDL-5 ApoB | 0.694999 | 0.612339 | 0.945104 | 0.935075 | 0.463062 | 0.586822 |
| LDL-6 ApoB | 0.11269 | 0.159085 | 0.403383 | 0.527792 | 0.001695 | 0.009154 |
| HDL-1 TG | 0.035964 | 0.065703 | 0.000562 | 0.008856 | 0.042035 | 0.104566 |
| HDL-2 TG | 0.066023 | 0.102526 | 0.003444 | 0.026363 | 0.537091 | 0.601229 |
| HDL-3 TG | 0.602409 | 0.548656 | 0.055887 | 0.129019 | 0.154823 | 0.30033 |
| HDL-4 TG | 0.216467 | 0.266255 | 0.441764 | 0.552495 | 0.000704 | 0.005457 |
| HDL-1 Chol | 0.449612 | 0.441931 | 0.836351 | 0.862687 | 0.374948 | 0.523149 |
| HDL-2 Chol | 0.168397 | 0.21567 | 0.035876 | 0.100342 | 0.186153 | 0.337918 |
| HDL-3 Chol | 0.005736 | 0.017802 | 0.006054 | 0.033864 | 0.652199 | 0.673923 |
| HDL-4 Chol | 0.000978 | 0.00486 | 0.017999 | 0.059494 | 0.380354 | 0.523149 |
| HDL-1 FC | 0.818772 | 0.70149 | 0.513672 | 0.602488 | 0.475333 | 0.596743 |
| HDL-2 FC | 0.313823 | 0.336207 | 0.535206 | 0.608128 | 0.043771 | 0.106906 |
| HDL-3 FC | 0.011914 | 0.029046 | 0.125951 | 0.226152 | 0.346861 | 0.510044 |
| HDL-4 FC | 0.006019 | 0.017802 | 0.355435 | 0.482522 | 0.035338 | 0.096876 |
| HDL-1 PL | 0.720396 | 0.630245 | 0.789355 | 0.831911 | 0.36071 | 0.510044 |
| HDL-2 PL | 0.266155 | 0.305603 | 0.245019 | 0.352828 | 0.768518 | 0.748079 |
| HDL-3 PL | 0.001555 | 0.006661 | 0.069348 | 0.147095 | 0.357246 | 0.510044 |
| HDL-4 PL | 0.000006 | 0.000105 | 0.009704 | 0.044105 | 0.027351 | 0.076543 |
| HDL-1 ApoA1 | 0.929222 | 0.774747 | 0.849332 | 0.869907 | 0.59114 | 0.645305 |
| HDL-2 ApoA1 | 0.006947 | 0.019178 | 0.183727 | 0.293359 | 0.10866 | 0.235424 |
| HDL-3 ApoA1 | 0.000964 | 0.00486 | 0.00584 | 0.033864 | 0.969497 | 0.866645 |
| HDL-4 ApoA1 | 0.000051 | 0.000638 | 0.007398 | 0.037102 | 0.083239 | 0.192785 |
| HDL-1 ApoA2 | >0.999999 | 0.801484 | 0.47867 | 0.574751 | 0.2872 | 0.458694 |
| HDL-2 ApoA2 | 0.974322 | 0.801484 | 0.689592 | 0.754092 | 0.559291 | 0.615816 |
| HDL-3 ApoA2 | 0.319347 | 0.336207 | 0.166018 | 0.274382 | 0.53054 | 0.601229 |
| HDL-4 ApoA2 | 0.034263 | 0.063529 | 0.102319 | 0.188371 | 0.38945 | 0.523149 |

**Supplementary Table S2: Characteristics of the ROC analysis for pyruvic acid, including the color-coded representation of the highest Youden Index.**

| **Pyruvic acid mmol/L** | **Sensitivity%** | **95% CI** | **Specificity%** | **95% CI** | **Likelihood ratio** | **Youden Index** |
| --- | --- | --- | --- | --- | --- | --- |
| > 0.01500 | 100.00 | 89.57% - 100.0% | 10 | 3.958% - 23.05% | 1.111 | 10.00 |
| > 0.03500 | 96.97 | 84.68% - 99.84% | 45 | 30.71% - 60.17% | 1.763 | 41.97 |
| > 0.04500 | 81.82 | 65.61% - 91.39% | 75 | 59.81% - 85.81% | 3.273 | 56.82 |
| > 0.05500 | 81.82 | 65.61% - 91.39% | 85 | 70.93% - 92.94% | 5.455 | 66.82 |
| > 0.06500 | 72.73 | 55.78% - 84.93% | 95 | 83.50% - 99.11% | 14.55 | 67.73 |
| > 0.07500 | 66.67 | 49.61% - 80.25% | 100 | 91.24% - 100.0% |  | 66.67 |
| > 0.08500 | 51.52 | 35.22% - 67.50% | 100 | 91.24% - 100.0% |  | 51.52 |
| > 0.09500 | 45.45 | 29.84% - 62.01% | 100 | 91.24% - 100.0% |  | 45.45 |
| > 0.1050 | 42.42 | 27.24% - 59.19% | 100 | 91.24% - 100.0% |  | 42.42 |
| > 0.1150 | 33.33 | 19.75% - 50.39% | 100 | 91.24% - 100.0% |  | 33.33 |
| > 0.1300 | 27.27 | 15.07% - 44.22% | 100 | 91.24% - 100.0% |  | 27.27 |
| > 0.1500 | 24.24 | 12.83% - 41.02% | 100 | 91.24% - 100.0% |  | 24.24 |
| > 0.1750 | 18.18 | 8.611% - 34.39% | 100 | 91.24% - 100.0% |  | 18.18 |
| > 0.1950 | 15.15 | 6.650% - 30.92% | 100 | 91.24% - 100.0% |  | 15.15 |
| > 0.2250 | 12.12 | 4.816% - 27.33% | 100 | 91.24% - 100.0% |  | 12.12 |
| > 0.2800 | 6.06 | 1.077% - 19.61% | 100 | 91.24% - 100.0% |  | 6.061 |
| > 0.4850 | 3.03 | 0.1554% - 15.32% | 100 | 91.24% - 100.0% |  | 3.03 |

**Supplementary Table S3: Medication at the time of sample collection of patients.** The detailed medication of patients with inflammatory bowel disease and other extraintestinal manifestations except of PSC (EIM), PSC patients with confirmed inflammatory bowel disease (PSC-IBD), and PSC patients without confirmed IBD is shown in Table 4.

| **Medication at the time of sample collection** | **EIM**  **(n=64)** | **PSC-IBD**  **(n=24)** | **PSC**  **(n=9)** |
| --- | --- | --- | --- |
| None | 4 |  |  |
| Anti-tumor necrosis factor (TNF) monotherapy | 19 |  |  |
| Vedolizumab monotherapy | 2 |  |  |
| Ustekinumab monotherapy | 13 |  |  |
| Mesalazine monotherapy | 2 |  |  |
| Corticosteroids monotherapy | 2 |  |  |
| Azathioprine monotherapy | 3 |  |  |
| Anti-TNF + Corticosteroids | 3 |  |  |
| Anti-TNF + Mesalazine | 2 |  |  |
| Anti- TNF + Ustekinumab | 1 |  |  |
| Anti-TNF + Corticosteroids + Methotrexate | 1 |  |  |
| Mesalazine + Corticosteroids | 2 |  |  |
| Vedolizumab + Azathioprine + Mesalazine | 1 |  |  |
| Ustekinumab + Corticosteroids | 5 |  |  |
| Ustekinumab + Mesalazine | 2 |  |  |
| Ustekinumab + Mesalazine + Corticosteroids | 2 |  |  |
| Ursodeoxycholic acid (UDCA) + Mesalazine |  | 6 |  |
| UDCA + Anti-TNF |  | 1 |  |
| UDCA + Mesalazine + Anti TNF |  | 1 |  |
| UDCA + Osteoporosis prophylaxis + Mesalazine |  | 4 |  |
| UDCA + Osteoporosis prophylaxis + Anti-TNF |  | 1 |  |
| UCDA + Osteoporosis prophylaxis + Mesalazine + Corticosteroids |  | 1 |  |
| UDCA + Osteoporosis prophylaxis + Mesalazine + Azathiprine + Fat-soluble vitamins |  | 1 |  |
| UDCA + Osteoporosis prophylaxis + Antibiotics + Sertalin |  | 1 |  |
| UDCA + Osteoporosis prophylaxis + Mesalazine + Azathioprine + Corticosteroids |  | 1 |  |
| UDCA + Osteoporosis prophylaxis + Antibiotics + Corticosteroids + Anti TNF |  | 1 |  |
| UDCA + Osteoporosis prophylaxis + Mesalazine + Vedolizumab |  | 1 |  |
| UDCA + Osteoporosis prophylaxis + Azathioprine + Vedolizumab |  | 1 |  |
| UDCA + Corticosteroids + Naltrexon |  | 1 |  |
| UDCA + Corticosteroids |  | 1 |  |
| UDCA + Osteoporosis prophylaxis + Colestyramine |  | 1 |  |
| UDCA + Osteoporosis prophylaxis + Antibiotics + Fat-soluble vitamins |  | 1 |  |
| UDCA monotherapy |  |  | 2 |
| Osteoporosis prophylaxis monotherapy |  |  | 1 |
| UDCA + Osteoporosis prophylaxis |  |  | 1 |
| UDCA + Osteoporosis prophylaxis + Corticosteroids |  |  | 1 |
| UDCA + Antibiotics + Colestyramine |  |  | 1 |
| UDCA + Osteoporosis prophylaxis + Corticosteroids + Fat-soluble vitamins |  |  | 1 |
| Osteoporosis prophylaxis + Antibiotics |  |  | 1 |
| Missing Data |  |  | 1 |

**Supplementary Table S3: P-values of metabolites and lipoproteins according to the comparison of therapy groups.** Quantified metabolites and lipoproteins analyzed using nuclear magnetic resonance (NMR) spectroscopy with p-values (unpaired Mann-Whitney test without correction for multiple testing) for the comparison of PSC patients taking UDCA vs. UDCA and mesalazine. UDCA vs. UDCA and biologicals. as well as UDCA and mesalazine vs. UDCA and biologicals.

|  | **UDCA vs. UDCA + mesalazine** | **UDCA vs. UDCA + biologicals** | **UDCA + mesalazine vs. UDCA + biologicals** |
| --- | --- | --- | --- |
|  | **p-value** | **p-value** | **p-value** |
| Ethanol | >0.999999 | >0.999999 | >0.999999 |
| Trimethylamine-N-oxide | 0.137255 | 0.461538 | >0.999999 |
| 2-Aminobutyric acid | 0.339461 | 0.730769 | >0.999999 |
| Alanine | 0.236551 | 0.944056 | 0.171946 |
| Asparagine | 0.673014 | 0.08683 | 0.103507 |
| Creatine | 0.464775 | 0.645688 | 0.987072 |
| Creatinine | 0.638575 | 0.466783 | 0.903604 |
| Glutamic acid | 0.315988 | 0.078089 | 0.939318 |
| Glutamine | 0.491516 | 0.386946 | 0.942712 |
| Glycine | 0.080191 | 0.0169 | 0.417582 |
| Histidine | 0.304896 | 0.474359 | 0.568116 |
| Isoleucine | 0.767345 | 0.119464 | 0.100032 |
| Leucine | 0.105706 | 0.045455 | 0.644796 |
| Lysine | 0.230047 | 0.289627 | 0.983759 |
| Methionine | 0.528846 | 0.373543 | 0.701842 |
| N.N-Dimethylglycine | 0.388889 | >0.999999 | >0.999999 |
| Ornithine | 0.520268 | 0.082751 | 0.087993 |
| Phenylalanine | 0.462921 | 0.27972 | 0.863365 |
| Proline | 0.805021 | 0.62704 | 0.149887 |
| Sarcosine | 0.842729 | >0.999999 | 0.514706 |
| Threonine | 0.035508 | 0.066434 | 0.476487 |
| Tyrosine | 0.649038 | 0.600233 | 0.727052 |
| Valine | 0.579563 | 0.702214 | 0.904493 |
| 2-Hydroxybutyric acid | >0.999999 | 0.461538 | 0.352941 |
| Acetic acid | 0.735577 | 0.396853 | 0.223578 |
| Citric acid | 0.045814 | 0.277972 | 0.473012 |
| Formic acid | 0.496292 | 0.88345 | 0.382595 |
| Lactic acid | 0.740699 | 0.467366 | 0.787169 |
| Succinic acid | 0.496732 | 0.020979 | 0.073853 |
| Choline | 0.496732 | >0.999999 | 0.514706 |
| 2-Oxoglutaric acid | 0.716912 | >0.999999 | 0.272059 |
| 3-Hydroxybutyric acid | 0.137569 | 0.050117 | 0.259615 |
| Acetoacetic acid | 0.32287 | 0.331002 | 0.598416 |
| Acetone | 0.250911 | 0.013986 | 0.263575 |
| Pyruvic acid | 0.058069 | 0.646853 | 0.08161 |
| D-Galactose | >0.999999 | >0.999999 | >0.999999 |
| Glucose | 0.808981 | 0.445221 | 0.788542 |
| Glycerol | 0.685458 | 0.730769 | >0.999999 |
| Dimethylsulfone | 0.937092 | >0.999999 | 0.272059 |
| TG | 0.246292 | 0.365967 | 0.660472 |
| Chol | 0.536011 | 0.730769 | 0.524887 |
| LDL | 0.438348 | 0.945221 | 0.462346 |
| HDL | 0.860106 | 0.945221 | 0.807531 |
| ApoA1 | 0.811055 | 0.945221 | 0.883646 |
| ApoA2 | 0.285445 | 0.365967 | 0.807531 |
| ApoB100 | 0.150892 | 0.051282 | 0.883646 |
| LDL/HDL | 0.285445 | 0.365967 | >0.999999 |
| ApoB100/ApoA1 | 0.217907 | 0.24026 | >0.999999 |
| ApoB Particles | 0.150892 | 0.051282 | 0.883646 |
| VLDL Particles | 0.374937 | 0.234266 | 0.732547 |
| IDL Particles | 0.150892 | 0.034965 | 0.660472 |
| LDL Particles | 0.21091 | 0.022145 | 0.883646 |
| LDL-1 Particles | 0.21091 | 0.013986 | 0.216063 |
| LDL-2 Particles | 0.724233 | 0.628205 | 0.961215 |
| LDL-3 Particles | 0.285445 | 0.137529 | 0.883646 |
| LDL-4 Particles | 0.438757 | 0.445221 | 0.732547 |
| LDL-5 Particles | 0.217289 | 0.730769 | 0.402715 |
| LDL-6 Particles | 0.67713 | 0.628205 | 0.180187 |
| VLDL TG | 0.425151 | >0.999999 | 0.350194 |
| IDL TG | 0.425151 | 0.945221 | 0.660472 |
| LDL TG | 0.104198 | 0.022145 | 0.036522 |
| HDL TG | 0.21091 | 0.022145 | 0.148998 |
| VLDL | 0.374937 | 0.628205 | 0.350194 |
| IDL | 0.285445 | 0.180653 | 0.883646 |
| LDL | 0.438348 | 0.945221 | 0.462346 |
| HDL | 0.860106 | 0.945221 | 0.807531 |
| VLDL FC | 0.425151 | 0.628205 | 0.301067 |
| IDL FC | 0.425151 | 0.365967 | >0.999999 |
| LDL FC | 0.21091 | 0.234266 | 0.883646 |
| HDL FC | 0.880468 | 0.945221 | 0.807531 |
| VLDL PL | 0.478947 | 0.945221 | 0.256141 |
| IDL PL | 0.21091 | 0.628205 | 0.462346 |
| LDL PL | 0.285445 | 0.365967 | 0.883646 |
| HDL PL | 0.791415 | 0.730769 | >0.999999 |
| HDL ApoA1 | 0.811055 | 0.835664 | 0.732547 |
| HDL ApoA2 | 0.387098 | 0.628205 | 0.961215 |
| VLDL ApoB | 0.374937 | 0.234266 | 0.732547 |
| IDL ApoB | 0.150892 | 0.034965 | 0.660472 |
| LDL ApoB | 0.21091 | 0.022145 | 0.883646 |
| VLDL-1 TG | 0.658999 | 0.628205 | 0.301067 |
| VLDL-2 TG | 0.285445 | 0.945221 | 0.404331 |
| VLDL-3 TG | 0.425151 | 0.730769 | 0.404331 |
| VLDL-4 TG | 0.658999 | 0.730769 | 0.350194 |
| VLDL-5 TG | 0.658999 | 0.5338 | 0.301067 |
| VLDL-1 Chol | 0.534974 | 0.945221 | 0.446752 |
| VLDL-2 Chol | 0.285445 | 0.5338 | 0.660472 |
| VLDL-3 Chol | 0.425151 | 0.730769 | 0.301067 |
| VLDL-4 Chol | 0.21091 | 0.234266 | 0.590821 |
| VLDL-5 Chol | 0.859697 | 0.945221 | 0.786361 |
| VLDL-1 FC | 0.912205 | 0.381119 | 0.446752 |
| VLDL-2 FC | 0.21091 | 0.234266 | 0.961215 |
| VLDL-3 FC | 0.374937 | 0.365967 | 0.524887 |
| VLDL-4 FC | 0.536011 | 0.365967 | 0.961215 |
| VLDL-5 FC | 0.868401 | 0.62704 | 0.503151 |
| VLDL-1 PL | 0.912205 | 0.596737 | 0.337589 |
| VLDL-2 PL | 0.425151 | 0.945221 | 0.350194 |
| VLDL-3 PL | 0.285445 | 0.835664 | 0.404331 |
| VLDL-4 PL | 0.478947 | 0.5338 | 0.524887 |
| VLDL-5 PL | 0.789027 | 0.62704 | 0.608274 |
| LDL-1 TG | 0.125943 | 0.004662 | 0.047673 |
| LDL-2 TG | 0.104198 | 0.004662 | 0.098255 |
| LDL-3 TG | 0.104198 | 0.008159 | 0.256141 |
| LDL-4 TG | 0.144356 | 0.013986 | 0.289431 |
| LDL-5 TG | 0.085344 | 0.022145 | 0.750485 |
| LDL-6 TG | 0.21091 | 0.365967 | 0.524887 |
| LDL-1 Chol | 0.285445 | 0.073427 | 0.462346 |
| LDL-2 Chol | 0.929801 | 0.835664 | 0.524887 |
| LDL-3 Chol | 0.596154 | 0.628205 | 0.807531 |
| LDL-4 Chol | 0.67713 | 0.628205 | 0.660472 |
| LDL-5 Chol | 0.384553 | 0.945221 | 0.461134 |
| LDL-6 Chol | >0.999999 | 0.234266 | 0.143261 |
| LDL-1 FC | 0.21091 | 0.034965 | 0.350194 |
| LDL-2 FC | 0.791415 | 0.945221 | 0.883646 |
| LDL-3 FC | 0.596154 | 0.312354 | 0.961215 |
| LDL-4 FC | 0.743401 | 0.835664 | 0.660472 |
| LDL-5 FC | 0.59518 | 0.945221 | 0.507919 |
| LDL-6 FC | 0.880468 | 0.137529 | 0.180187 |
| LDL-1 PL | 0.21091 | 0.034965 | 0.216063 |
| LDL-2 PL | 0.791415 | 0.945221 | 0.660472 |
| LDL-3 PL | 0.363342 | 0.276807 | 0.941984 |
| LDL-4 PL | 0.552068 | 0.5338 | 0.608274 |
| LDL-5 PL | 0.337387 | 0.835664 | 0.523432 |
| LDL-6 PL | 0.723573 | 0.25 | 0.094538 |
| LDL-1 ApoB | 0.21091 | 0.013986 | 0.216063 |
| LDL-2 ApoB | 0.724233 | 0.628205 | 0.961215 |
| LDL-3 ApoB | 0.285445 | 0.137529 | 0.883646 |
| LDL-4 ApoB | 0.438757 | 0.445221 | 0.732547 |
| LDL-5 ApoB | 0.217289 | 0.730769 | 0.402715 |
| LDL-6 ApoB | 0.67713 | 0.628205 | 0.180187 |
| HDL-1 TG | 0.150892 | 0.022145 | 0.121526 |
| HDL-2 TG | 0.411953 | 0.073427 | 0.127666 |
| HDL-3 TG | 0.791415 | 0.137529 | 0.038623 |
| HDL-4 TG | 0.579594 | 0.385781 | 0.787169 |
| HDL-1 Chol | 0.613468 | 0.730769 | 0.732547 |
| HDL-2 Chol | 0.724233 | 0.835664 | >0.999999 |
| HDL-3 Chol | 0.411953 | 0.5338 | 0.789108 |
| HDL-4 Chol | 0.17911 | 0.137529 | 0.524887 |
| HDL-1 FC | 0.811055 | 0.945221 | 0.590821 |
| HDL-2 FC | 0.929801 | 0.864219 | 0.904493 |
| HDL-3 FC | 0.285445 | 0.234266 | 0.883646 |
| HDL-4 FC | 0.125943 | 0.180653 | 0.732547 |
| HDL-1 PL | 0.613468 | 0.5338 | 0.902634 |
| HDL-2 PL | 0.929801 | 0.628205 | 0.807531 |
| HDL-3 PL | 0.493621 | >0.999999 | 0.462346 |
| HDL-4 PL | 0.328306 | 0.180653 | 0.350194 |
| HDL-1 ApoA1 | 0.552068 | 0.628205 | 0.883646 |
| HDL-2 ApoA1 | 0.724233 | 0.5338 | 0.961215 |
| HDL-3 ApoA1 | 0.374937 | 0.628205 | 0.807531 |
| HDL-4 ApoA1 | 0.17911 | 0.137529 | 0.350194 |
| HDL-1 ApoA2 | 0.295375 | 0.234266 | 0.961215 |
| HDL-2 ApoA2 | 0.658999 | 0.345571 | >0.999999 |
| HDL-3 ApoA2 | 0.774164 | 0.601399 | 0.524887 |
| HDL-4 ApoA2 | 0.21091 | 0.180653 | 0.660472 |

**Supplementary Figure S1: Receiver Operating Characteristic (ROC) curve of the Partial Least Squares Discriminant Analysis (PLS-DA) for the comparison between PSC, EIM, and HC.** The curve and values of the area under the curve (AUC) for calibration (blue) and cross-validation (green) are provided.

**Supplementary Figure S2: Results of the Random Forest analysis with cross-validation.** For feature selection, the methods Mean Decrease in Impurity (MDI) and Mean Decrease Accuracy were applied. The parameters listed are those contributing to the discrimination between groups in both methods, thus having a score > 0.01 for MDI and a score > 0 for Mean Decrease Accuracy.

**Supplementary Figure S3: Comparison of metabolites and lipoproteins according to therapy group.** Metabolites and lipoproteins of patients with primary sclerosing cholangitis (PSC) according to their therapy status depicted as heat map **(A)** and column charts **(B)**: ursodeoxycholic acid (UDCA) (red, n=7), UDCA and mesalazine (blue, n=11) and UDCA and biologicals (green, n=6). The multiple t-test performed with FDR as a correction factor did not yield statistically significant results. Therefore, a t-test without correction for multiple tests was performed to show possible trends in altered metabolites and lipoproteins between the therapy groups. In **S3A** p-values ≤ 0.05 are blue and p-values > 0.05 are red. Most of the analyzed metabolites and lipoproteins have p values > 0.05 without statistical significance and without trends to possible alterations. Tendencies of correlations were found for LDL-1 TG, LDL-2 TG, and LDL-3 TG in the group receiving UDCA monotherapy compared with therapy with UDCA and biologicals (p <0.05). Associations with higher LDL-1 to LDL-3 TG levels were noted with UDCA monotherapy compared with UDCA and biologicals **(S3B)**.
