# Supplementary figures and images for: Distinct metabolomic and lipidomic profiles in serum samples of patients with primary sclerosing cholangitis
Source: Front Med (Lausanne). 2024 Jun 4;11:1334865. doi: 10.3389/fmed.2024.1334865 (PMC11184724; doi:10.3389/fmed.2024.1334865)

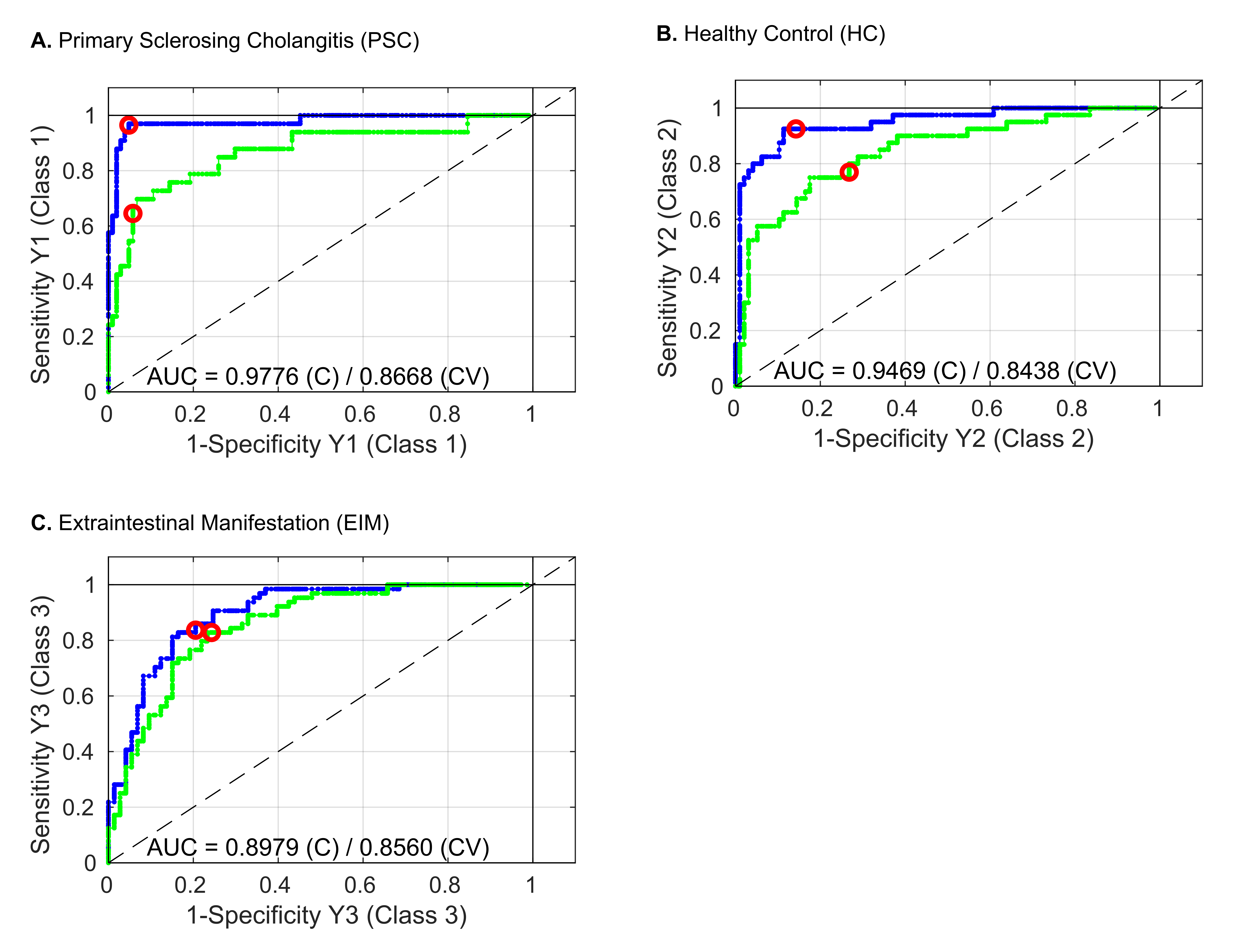

Supplement: Supplementary file 2 [file Image_1.PNG]

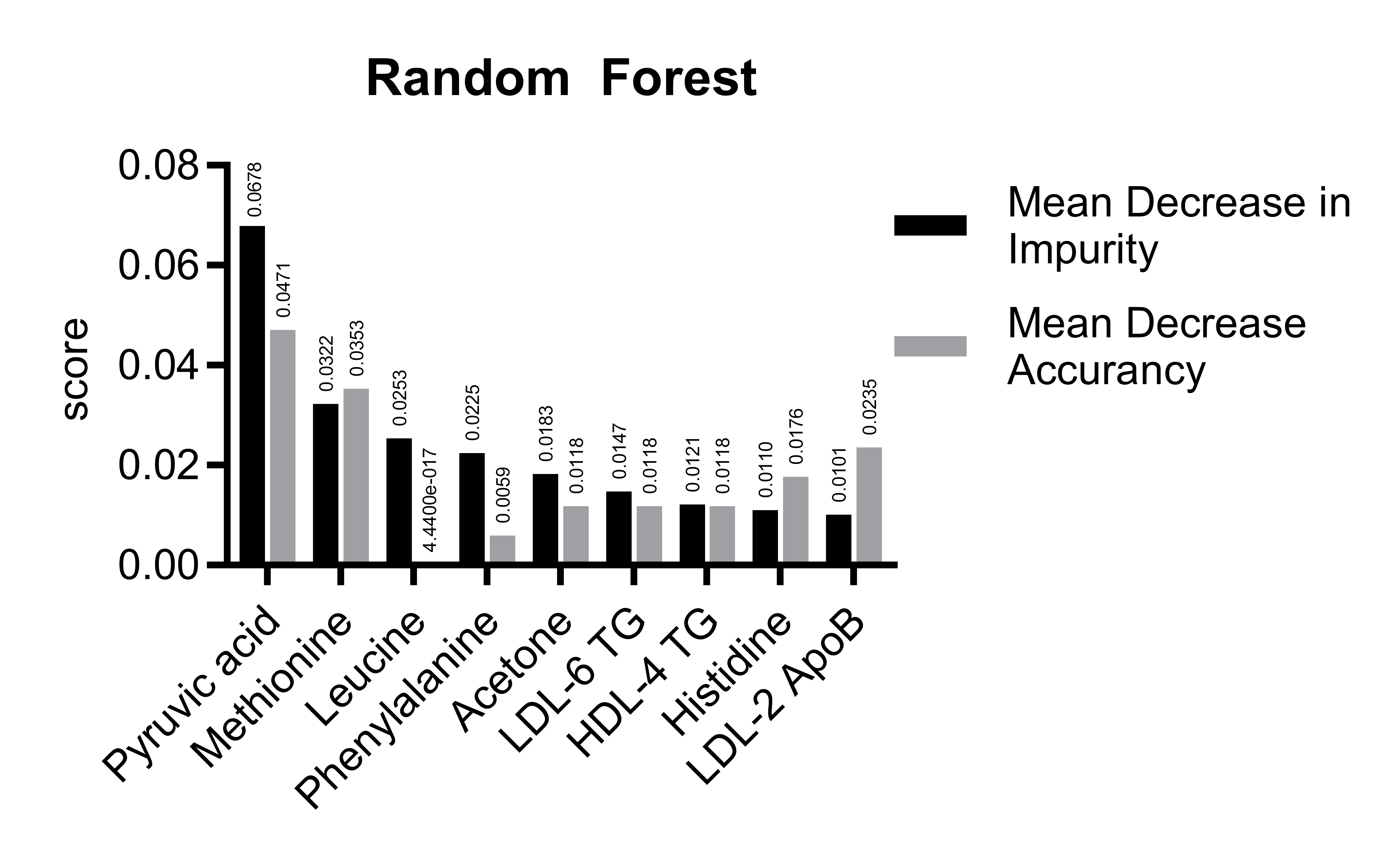

Supplement: Supplementary file 3 [file Image_2.PNG]

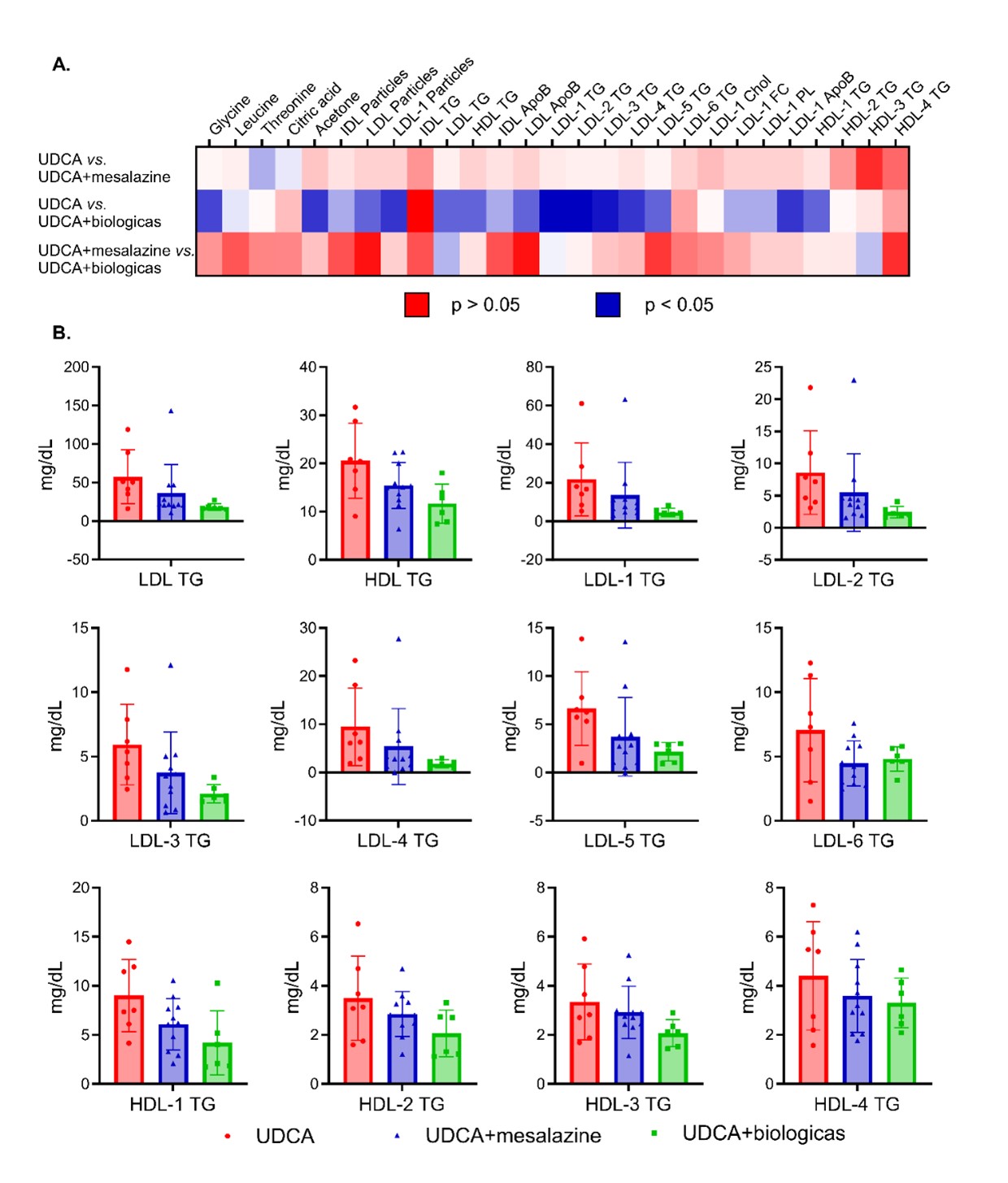

Supplement: Supplementary file 4 [file Image_3.JPEG]
